# Single-Cell Transcriptional Heterogeneity of Lymphatic Endothelial Cells in Normal and Inflamed Murine Lymph Nodes

**DOI:** 10.3390/cells10061371

**Published:** 2021-06-02

**Authors:** Eliane Sibler, Yuliang He, Luca Ducoli, Nadja Keller, Noriki Fujimoto, Lothar C. Dieterich, Michael Detmar

**Affiliations:** 1 Institute of Pharmaceutical Sciences, Swiss Federal Institute of Technology, ETH Zurich, Vladimir-Prelog-Weg 3, 8093 Zürich, Switzerland; eliane.sibler@pharma.ethz.ch (E.S.); yuliang.he@pharma.ethz.ch (Y.H.); luca.ducoli@pharma.ethz.ch (L.D.); nadkelle@student.ethz.ch (N.K.); lothar.dieterich@pharma.ethz.ch (L.C.D.); 2 Department of Dermatology, Shiga University of Medical Science, Otsu 520-2192, Japan; noriki@belle.shiga-med.ac.jp

**Keywords:** scRNA-seq, inflammation, lymph node, lymphatic endothelial cells

## Abstract

The lymphatic system plays a crucial role in immunity and lymph nodes (LNs) undergo drastic remodeling during inflammation. Here, we used single-cell RNA sequencing to investigate transcriptional changes in lymphatic endothelial cells (LECs) in LNs draining naïve and inflamed skin. We found that subsets of LECs lining the different LN sinuses responded individually to skin inflammation, suggesting that they exert distinct functions under pathological conditions. Among the genes dysregulated during inflammation, we confirmed an up-regulation of CD200 in the LECs lining the subcapsular sinus floor with a possible function in immune regulation. Furthermore, by in silico analysis, we predicted numerous possible interactions of LECs with diverse immune cells in the LNs and found similarities in the transcriptional changes of LN LECs in different skin inflammation settings. In summary, we provide an in-depth analysis of the transcriptional landscape of LN LECs in the naïve state and in skin inflammation.

## 1. Introduction

The lymphatic system transports fluid, proteins, and cells from peripheral tissues back to the blood circulation. In doing so, it also transports both antigens and tissue-derived antigen-presenting cells to the regional draining lymph nodes (LNs), where they interact with lymphocytes. In addition, lymphatic endothelial cells (LECs) in LNs have been shown to express self-antigens to present them to T lymphocytes directly and to regulate their functions via the expression of immune-regulatory molecules such as PD-L1 [1,2]. Consequently, the lymphatic system is essential for maintaining peripheral tolerance and the mounting of efficient immune responses [3].

Lymphatic sinuses in LNs are lined with highly specialized LECs, such as the cells lining the subcapsular sinus (SCS) as well as the medullary sinuses, which are essential for LN architecture and organization [4,5]. Although overlooked for a long time, LECs have diverse functions beyond their role as the structural cells of the lymphatic system. LECs have been found to facilitate immune cell migration into lymphatics and to draining LNs via the expression of chemokines and adhesion molecules. LECs in the LN form the barrier between the afferent lymph and the LN parenchyma, providing specific migration and transport routes into and out of the LN for diverse lymph-borne molecules and cells. LN LECs have also been reported to actively partake in antigen presentation by sequestering, storing, and releasing antigens originating from peripheral tissues [6,7], but their molecular and functional heterogeneity has been difficult to study for a long time, at least in part due to their low abundance. Recent advances in next-generation sequencing technologies, especially the development of single-cell sequencing platforms, provide a means to analyze such cell types with low abundance in tissues much more closely. This has led to the discovery of substantial heterogeneity within the LN LECs, both regarding gene expression and functions [8,9,10].

Inflammatory responses elicited in peripheral tissues are transmitted by and reflected in the lymphatic vasculature, locally and in draining LNs. For instance, inflammation leads to pronounced lymphangiogenesis in draining LNs mediated by vascular endothelial growth factor A (VEGFA) [11,12,13], whereas T cell-derived IFN-γ has been shown to decrease LN lymphangiogenesis, while triggering transcriptional responses, including up-regulation of CD41 [14,15]. Imiquimod is a small-molecule immune stimulator that directly activates Toll-like receptor 7 (TLR7) [16]. Topical application of imiquimod in mice results in skin inflammation that largely resembles psoriasis in humans, in terms of histological features as well as transcriptomic signatures [17,18]. Here, we used the imiquimod model to examine the transcriptional responses of LN LECs to skin inflammation by single-cell RNA sequencing (scRNA-seq). We identified dysregulated gene sets specific to individual LN LEC subpopulations at discrete anatomical locations, indicating their differential reactions to inflammation. Furthermore, we showed that CD200 was up-regulated in LN LECs lining the SCS floor at the protein level and predicted potential interactions between LECs and other cells in the LNs. Moreover, we found a substantial overlap in the LEC transcriptional responses to different types of inflammation, suggesting that the functions of LEC subsets in LNs may be conserved in various pathological conditions.

## 2. Materials and Methods

### 2.1. Animals

C57BL/6NRj mice were acquired from Janvier Labs and kept in an SOPF facility. All animal experiments were performed in agreement with national guidelines (Swiss Animal Experimentation Ordinance) and were approved by the veterinary authorities (Kantonales Veterinäramt Zürich, licenses 212/16 and 244/19).

### 2.2. Inflammation Mouse Model

Skin inflammation resembling psoriasis was induced in mouse ears by topical application of imiquimod-containing cream (Aldara, 5% imiquimod, MEDA GmbH, Wangen-Brüttisellen, Switzerland). The ears of 8-week-old female C57BL/6 mice were shaved, and Aldara cream was applied to both sides of the ears daily for 7 days under isoflurane anesthesia. On day 7, the mice were sacrificed and the auricular LNs were dissected for sequencing or embedding in OCT.

### 2.3. Isolation of LECs from Lymph Nodes

Auricular lymph nodes were dissected directly after sacrifice and digested, essentially as previously described [19]. Briefly, the capsule was disrupted by ripping it with a needle. The LNs were transferred to 1 mL digestion medium per 25 mg tissue, consisting of 0.8 mg/mL Dispase II (04942078001, Roche, Basel, Switzerland), 0.2 mg/mL Collagenase I (LS004197, Worthington, Lakewood, NJ, USA), and 0.1 mg/mL DNase I (10104159001, Roche) in DMEM (41965-039, Gibco, Thermo Fisher Scientific, Waltham, MA, USA). They were incubated at 37 °C for 20 min, with gentle agitation by inverting the tube every 5 min. Thereafter, the LNs were gently disrupted with a pipette, and after letting the tissue pieces settle to the bottom the supernatant was removed. The LNs were incubated at 37 °C again with fresh digestion medium for 10 min and then disrupted by pipetting for 30 s. The supernatant was removed and the remaining LN fragments incubated at 37 °C with fresh digestion medium. The fragments were mixed with a pipette every 5 min until all were digested. This last fraction of digested LN contains the highest frequency of LECs and was thus used for sorting. Cells were washed in FACS buffer (1% FBS and 2 mM EDTA) and stained with antibodies for CD45.2 FITC (553772, BD Biosciences, San Diego, CA, USA), CD31 APC (551262, BD Biosciences) and PDPN PE (12-5381-82, eBioscience, Thermo Fisher Scientific). ZombieNIR (423106, BioLegend, San Diego, CA, USA) was added to exclude dying cells. Single live LECs (CD45^−^CD31^+^PDPN^+^) were sorted directly into lysis buffer containing 0.1% Triton X-100, 2.5 mM dNTPs, 2.5 μM oligo-dT, 1 U/μL RNasin Plus RNase inhibitor (Promega, Madison, WI, USA) in 384-well plates with the BD FACS Aria II using FACS Diva 6.1.3. The plates were stored at −80 °C until further processing. Library preparation using a miniaturized version of the Smart-seq2 protocol and sequencing were done at the Functional Genomics Center Zürich (FGCZ) [9,20]. cDNA libraries were sequenced with Illumina HiSeq2500 using single-read 125-bp chemistry at a depth of 700,000 reads per cell on average.

### 2.4. Single-Cell RNA-Seq Data Processing

The Nextera adapter sequences and low-quality bases were removed using Trimmomatic v0.33 [21]. Trimmed reads were aligned to the Ensembl mm10 mouse reference genome (release 92) using STAR v2.4.2a [22]. Gene expression quantification was computed with the “featureCounts” function in the Rsubread package v1.26.1 [23]. Quality filtering was performed with the Scran package v1.4.5; cells with library size or feature size 3 median absolute deviations (MADs) away from the median or with mitochondrial contents 3 MADs above the median were dropped as outliers [24]. Genes expressed in at least 15% of the cells were grouped in accordance with their count–depth relationship using SCnorm v0.99.7, which applied a quantile regression within each group to estimate scaling factors and normalize for sequencing depth [25]. Cells with detected *Cd45* expression were removed prior to downstream analyses.

Counts were processed with Seurat 3.1.5 [26,27]. Cells with <400,000 or >500,000 counts, as well as cells with >10% mitochondria or ribosomal reads, were excluded for quality control. Counts were log-normalized and the top 2000 variable features were identified with the “NormalizeData” and “FindVariableFeatures” functions. Datasets were integrated (1400 anchor features) with “FindIntegrationAnchors” and “IntegrateData” to correct for batch effects. The cell cycle phase (G1, S, G2/M) of each cell was defined by the “CellCycleScoring” function, based on the expression of a core set of cell cycle-related genes [28]. The cell cycle effects were regressed out using “ScaleData”. Principal component analysis (PCA) was performed with “RunPCA.” Cell clusters were identified with “FindClusters” (PCA dims = 20; resolution = 0.5) based on the shared nearest neighbor (SNN) graph constructed with “FindNeighbors” and their marker genes were determined with “FindConservedMarkers.” Uniform manifold approximation and projection (UMAP) from “RunUMAP” was used for data visualization.

### 2.5. Differential Gene Expression and Gene Ontology Analysis

Differential expression (DE) analysis comparing the imiquimod versus control samples was performed with “FindMarkers” (min.pct = 0.2, avg_logFC > 0.2, p_val_adj < 0.05) for all three clusters. Gene ontology analysis of biological processes enriched for DE genes was performed with a PANTHER overrepresentation test (GO Ontology database, doi:10.5281/zenodo.4495804, released 2021-02-01, GO biological process complete, Fisher’s exact test) [29,30,31]. Genes expressed in both naïve and inflamed data were used as background. Only GO terms with *p* value < 0.05 were used for further analysis.

### 2.6. RNA Velocity

The RNA velocity analysis of naïve and inflamed samples was performed as previously described [32]. In a first step, spliced and unspliced counts were calculated from sorted aligned bam files of the 559 single cells using the velocyto.py run-smartseq2 function with default settings. From here, we followed the velocyto.R manual (http://velocyto.org/, accessed on 30 April 2021) and used spliced (emat) and unspliced (nmat) to estimate the RNA velocity of naïve and inflamed data combined. We restricted our analysis to protein-coding genes to avoid nuclear or cytoplasmic RNA retention biases. Using the cell-type annotation defined in Section 2.5, we performed gene filtering using the min.max.cluster.average parameter set to 5 and 1 for emat and nmat, respectively. RNA velocity was then estimated with the function gene.relative.velocity.estimates with default parameters except for kCells = 5 and fit.quantile = 0.5. To select differentially “speeded” (DS) genes, we first calculated the RNA velocity values using the formula

(1) $$\left[\frac{dS}{dt}\right]=\beta \cdot U_{g}\left(t_{c}\right)-\gamma_{g}\cdot S_{g}\left(t_{c}\right),$$

where β is equal to 1, $\gamma_{g}$ is the estimated gamma coefficient ($gamma), and $S_{g}\left(t_{c}\right)$ and $U_{g}\left(t_{c}\right)$ are the normalized emat ($conv.emat.norm) and nmat ($conv.nmat.norm) values of a gene g in a cell $t_{c}$, respectively. After plotting the mean RNA velocity per gene between naïve and inflamed conditions, we calculated the perpendicular distance between each point and the slope = 1. Finally, we selected DS genes having a slope distance higher than 0.1. Gene ontology for biological processes and pathway analyses of DS were performed as described above, using protein-coding genes expressed in both conditions as background.

### 2.7. Intercellular Interactions in Naïve Lymph Nodes

Whole LN scRNA-seq data were downloaded from GSE139658 (GSM4145642, GSM4145645 and GSM4145648), where the cell populations were isolated from inguinal LNs and depleted for CD3 and CD19 [33]. Datasets were integrated for unsupervised clustering analysis and cell types were assigned based on marker expression: B cells (*Cd19^+^*), CD8 T cells, macrophages (*Csf1r^+^Cd68^+^*), conventional dendritic cells cDC1 (*Itgax^+^Xcr1^+^Clec9a^+^*), CD69^+^ T cells (Cd69_T), migratory dendritic cells (*Ccr7^+^MHCII^+^*), NK cells (*Ncr1^+^*), CD4 T cells, endothelial cells (*Cd31^+^*), fibroblasts (*Pdgfrb^+^Col1a1^+^*) and neutrophils (*Cd3^−^Cd19^−^Itgam^+^Cd33^+^*). Mouse gene symbols were converted to human orthologs using the biomaRt package, and CellPhoneDB v2.0 (*p* value 0.05, threshold 0.1) was subsequently used to uncover potential ligand–receptor interactions between LN LECs and other LN cell types [34,35]. Significant interaction pairs were selected, from which ligand or receptor molecules on LN LECs were further intersected with our differentially expressed gene lists from the imiquimod dataset.

### 2.8. Comparison to Inflammatory Responses in LN LECs under Cutaneous Oxazolone Challenge

ScRNA-seq data were downloaded from GSE145121 and reanalyzed in Seurat. Cells with fewer than 100 genes detected and genes expressed in fewer than three cells were excluded as previously described [10]. The top 2000 variable features were identified; the control and oxazolone datasets were integrated using 1400 anchors. The effects of cell cycle, library size and feature number were regressed out. Unsupervised clustering was performed and different LN LEC subtypes were denoted by their respective markers (e.g., *Ackr4*, *Lyve1*, *Madcam1*, and *Mrc1*). Differentially expressed genes in the respective LN LEC subtype between the control and oxazolone datasets were defined (min.pct = 0.25, logfc.threshold = 0.25, and *p*_val_adj < 0.05).

### 2.9. Immunofluorescence Staining

Auricular LNs were embedded in OCT and frozen in liquid nitrogen. Then, 7-µm sections were cut with a cryostat and fixed with acetone and 80% methanol. PBS containing 0.2% BSA, 5% donkey serum, 0.3% Triton-X100, and 0.05% NaN_3_ was used for blocking for 1 h at room temperature. The sections were then stained with primary antibodies diluted in blocking solution at 4 °C overnight. The antibodies used were rabbit anti-mouse LYVE1 (11-034, AngioBio, San Diego, CA, USA, 1:600), goat anti-mouse LYVE1 (AF2125, R&D Systems, Minneapolis, MN, USA, 1:100), rat anti-mouse CD200 (BE0299, BioXCell, Lebanon, NH, USA, final concentration 3 µg/mL), and rabbit anti-mouse ANXA2 (ab178677, Abcam, Cambridge, UK, 1:400). After secondary staining with donkey anti-rat Alexa 488 (A-21208, Thermo Fisher Scientific), donkey anti-goat Alexa 594 (A-11058, Thermo Fisher Scientific), donkey anti-rabbit Alexa 594 (A-21207, Thermo Fisher Scientific), or donkey anti-rabbit Alexa 488 (A-21206, Thermo Fisher Scientific) and Hoechst 33342 (H3570, Thermo Fisher Scientific), slides were mounted with Mowiol. Images were taken with an Axioskop 2 Mot Plus equipped with AxioCam MRc (Carl Zeiss, Oberkochen, Germany) or an LSM 780 upright confocal microscope (Carl Zeiss). Quantification was performed with Fiji by outlining the area of interest, selecting the LYVE1^+^ area with “Analyze Particles,” and measuring intensity in the ANXA2 image with “Measure” [36].

### 2.10. FACS Analysis of Freshly Isolated LN LECs

Auricular LNs were dissected from mice with and without skin inflammation on day 6 and digested as previously described [15]. The capsule was disrupted with two 25G needles and the LNs incubated for 20 min at 37 °C in digestion medium consisting of DMEM with 2% FCS, 1.2 mM CaCl_2_, 40 µg/mL DNase I, and 1 mg/mL Collagenase IV (17104-019, Gibco). After incubation, the supernatant was removed and the LN fragments washed once with DMEM. The fragments containing the LN stromal cells were then incubated again at 37 °C for 15 min in 750 µL digestion medium containing 3.5 mg/mL Collagenase IV. The remaining fragments were then disrupted by pipetting up and down 2 × 100 times, with addition of 5 mM EDTA. The cell suspension was filtered through a 40 µm cell strainer, washed and treated with Fc-block (rat anti-mouse CD16/32, 101302, BioLegend) in FACS buffer (DPBS with 1% FBS, 2 mM EDTA and 0.02% NaN_3_) for 20 min. Cells were stained with rat anti-mouse CD45 APC-Cy7 (103116, BioLegend), rat anti-mouse CD31 APC (551262, BD Biosciences), hamster anti-mouse podoplanin PE (25-5381-82, eBioscience), rat anti-mouse CD200 PE-Dazzle594 (123820, BioLegend) and ZombieNIR diluted in PBS. The cells were then fixed with the Anti-Mouse/Rat Foxp3 PE staining set (72-5775-40, eBioscience) according to the kit’s instructions, and data were acquired on a BD LSRFortessa.

### 2.11. Statistical Analysis

Statistical analysis was performed with Prism 9.0.0 (GraphPad, San Diego, CA, USA). Unpaired two-tailed Student’s *t*-test was used for comparisons of two groups, *p* values ≤ 0.05 were considered significant (indicated in plots as *p* ≤ 0.05 *, *p* ≤ 0.01 **, *p* ≤ 0.001 ***).

## 3. Results

### 3.1. Characterization of LN LEC Subtypes by Single-Cell RNA Sequencing

**Figure 1:**
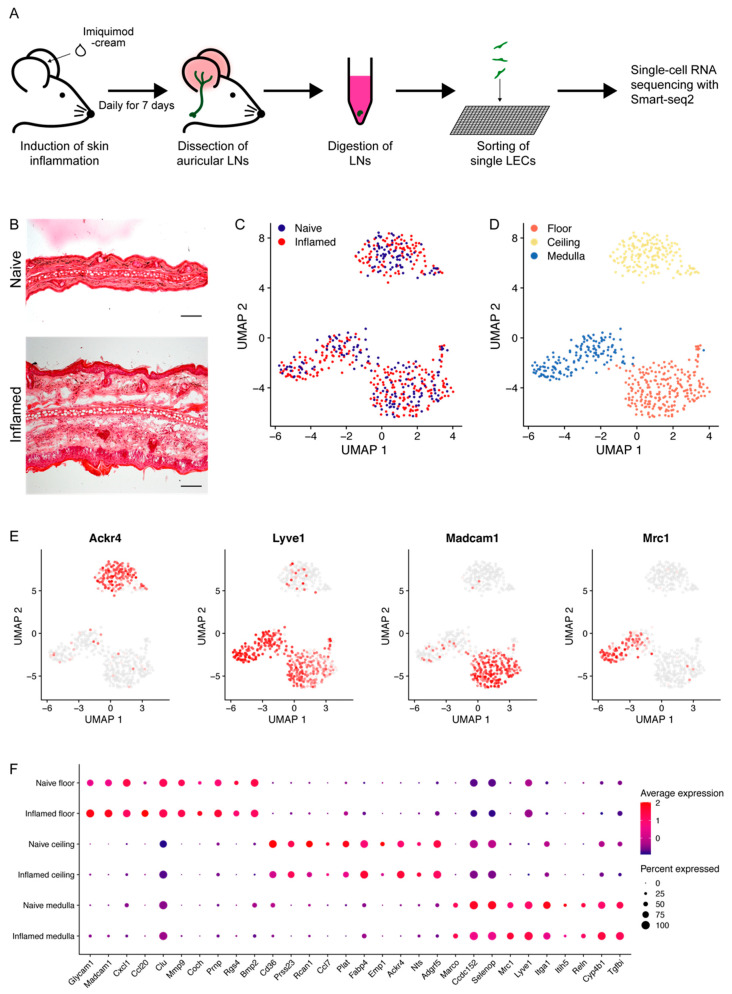
Single-cell RNA sequencing of LECs from LN draining healthy and imiquimod-inflamed skin and clustering into three subpopulations. (**A**) Schematic representation of the experimental workflow. (**B**) Representative H&E staining of sections from naïve and inflamed ears. Scale bar 100 µm. (**C**) UMAP with all 229 naïve and 330 inflamed LN LECs colored according to condition. (**D**) UMAP with all cells colored by LN LEC subsets as found through unsupervised clustering. (**E**) Expression of known marker genes for different LN LEC subsets. *Ackr4* marks the SCS ceiling, *Lyve1* both the SCS floor and the medullary sinus, *Madcam1* the SCS floor, and *Mrc1* the medullary sinus. (**F**) Dot plot of the top 10 (ranked by *p* values) conserved markers for each of the three clusters, indicating their expression in naïve and inflamed conditions.

LNs undergo drastic changes in the context of inflammation. To study in detail the changes LECs undergo during inflammation, we employed scRNA-seq technology (Figure 1A). First, we induced skin inflammation in mouse ears by topical application of imiquimod. After 7 days, when the ears became significantly swollen (Figure 1B), we harvested the auricular LNs that drain the affected area and digested them to generate a single-cell suspension. Then, we isolated single-LN LECs, defined as CD45^−^CD31^+^PDPN^+^ cells, by FACS and finally subjected them to scRNA-seq using the Smart-seq2 method (Figure 1A,B, Supplementary Materials Figure S1A). 

A total of 229 naïve and 330 inflamed cells remained after quality control (see the Materials and Methods section) and were used for downstream analyses. Unsupervised clustering with Seurat identified three subsets of LN LECs, which were equally represented in naïve and inflamed LECs (Figure 1C,D) [26,27]. All three clusters showed robust expression of the pan-endothelial marker *Cd31* (also known as platelet and endothelial cell adhesion molecule, *Pecam1*), as well as the lymphatic markers *Pdpn* (podoplanin), *Prox1* (prospero homeobox protein 1) and *Vegfr3* (vascular endothelial growth factor receptor 3, also called fms-related tyrosine kinase 4, *Flt4*), confirming the LEC identity of the profiled cells (Supplementary Materials Figure S1B). The marker genes *Ackr4* (atypical chemokine receptor 4), *Lyve1* (lymphatic vessel endothelial hyaluronan receptor 1), *Madcam1* (mucosal vascular addressin cell adhesion molecule 1), and *Mrc1* (mannose receptor C-type), which have been known to be expressed in specific LN LEC subtypes residing in defined anatomical locations [9,10,37], allowed us to assign these clusters to LECs lining the SCS floor (floor LECs), the SCS ceiling (ceiling LECs) and the medullary sinuses (medullary LECs) (Figure 1D,E). The abundance of the three LEC subsets remained fairly comparable in the naïve state and under inflammation (38.9% resp. 43.0% floor, 34.5% resp. 30% ceiling and 26.6% resp. 27% medullary LECs) (Supplementary Materials Figure S1C). Furthermore, we found a very similar distribution to the cell cycle phases in naïve and inflamed conditions (Supplementary Materials Figure S1D).

Among the top 10 markers of the floor LEC cluster, some have previously been reported as SCS floor LEC-specific genes, such as *Glycam1* (glycosylation-dependent cell adhesion molecule 1), *Madcam1*, and *Ccl20* [8,9,19,38] (Figure 1F). Also, the floor LECs further expressed *Itga2b* (integrin alpha 2b), *Cd44*, *Coch* (cochlin) and *Lyve1* [9,39]. By contrast, SCS ceiling LECs expressed *Ackr4*, yet lacked *Lyve1* (Figure 1E,F) [37]. While medullary LECs also expressed *Lyve1*, they lacked the expression of most of the above-mentioned floor markers, but instead expressed *Mrc1*, *Marco* (macrophage receptor with collagenous structure) and *Reln* (Reelin) [9,10]. In conclusion, the subpopulations of LN LECs we identified here have shown the expected marker gene expression that corresponds well with previous reports.

### 3.2. LN LEC Subtypes Display Distinct Gene Expression in Inflammation

**Figure 2:**
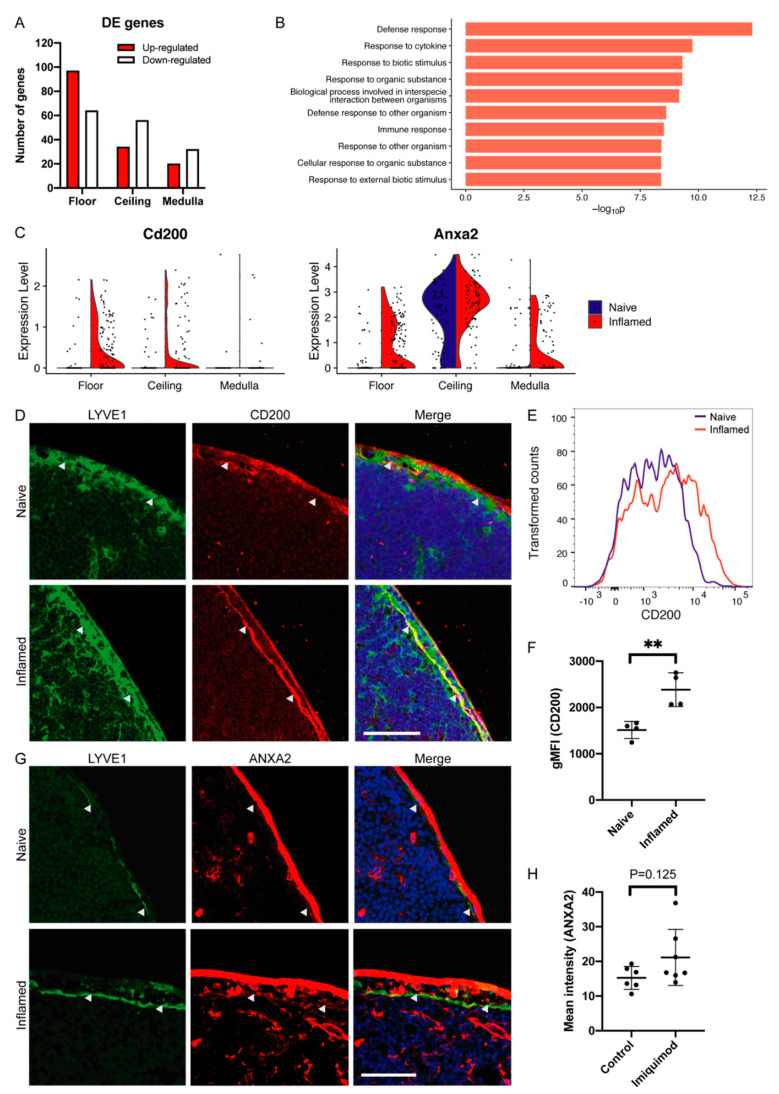
Differential gene expression between LN LECs from naïve and inflamed conditions. (**A**) The number of genes up- and down-regulated in inflammation versus naïve conditions in each LEC subset (log_2_FC > 0.2 and adjusted *p* < 0.05). (**B**) Top 10 GO terms of biological processes enriched for genes differentially expressed in inflammation in floor LECs (*p* < 0.05, ranked by *p* values). (**C**) Violin plots showing up-regulation of *Cd200* and *Anxa2* in the floor LECs during inflammation. (**D**) Representative immunofluorescence images of CD200 in LNs from naïve and inflamed conditions. Arrowheads: SCS floor, scale bar 50 µm. (**E**) FACS plot showing a representative histogram of CD200 expression on LECs, from naïve and inflamed LNs. (**F**) Expression of CD200 by LN LECs under naïve and inflamed conditions measured by FACS (*n* = 4). Mean + SD, *p* ≤ 0.01 **. (**G**) Representative immunofluorescence images of ANXA2 in LNs from naïve and inflamed conditions. Arrowhead: SCS floor, scale bar 50 µm. (**H**) Quantification of ANXA2 expression in floor LECs in LN sections, mean intensity of ANXA2 staining in LYVE1^+^ area averaged over 2–5 images per replicate (*n* = 6 naïve resp. 7 inflamed). Mean + SD.

To investigate the transcriptional responses of the LEC subsets lining the skin-draining LNs to skin inflammation, we performed differential gene expression (DE) analysis comparing the healthy and inflamed conditions. We identified DE genes (log_2_FC > 0.2 and adjusted *p* < 0.05) in all three subsets of LECs separately (Supplementary Materials Figure S2A and Table S1). Interestingly, the LEC subset with the highest number of up- and down-regulated genes was the floor (161 genes), while more modest alterations were found in the SCS ceiling (90 genes) and medulla (52 genes) (Figure 2A). Among the top DE genes in each subpopulation, we found several genes that have previously been associated with inflammation, such as the chemokine *Ccl20*, the adhesion molecule *Glycam1* and the extracellular matrix component *Fn1* (fibronectin 1) [40,41,42,43] (Supplementary Materials Figure S2A), suggesting that LECs contribute to or regulate inflammatory processes in the draining LNs.

Remarkably, a comparison of the DE gene sets from the three LN LEC subsets showed only a minor overlap, indicating that LN LECs react to inflammatory stimuli disparately depending on their localizations and functions (Supplementary Materials Figure S2B). Using gene ontology (GO) analysis, we aimed to gain insights into what biological processes might be underlying the observed gene expression patterns. DE genes of both the SCS floor and ceiling LECs were enriched in GO terms related to immune response, defense response, response to other organisms and response to cytokines (Figure 2B, Supplementary Materials Figure S2C and Table S2). On the other hand, genes differentially expressed in medullary LECs were mainly associated with response to stimulus and metabolic processes (Supplementary Materials Figure S2C and Table S2). In summary, these data highlight the distinct territorial and functional responses of LN LECs to inflammation in upstream tissues.

### 3.3. Specific Up-Regulation of Cd200 and Anxa2 in Floor LECs during Inflammation

To confirm our sequencing results at the protein level, we selected two genes, *Cd200* and *Anxa2*, which were both induced in floor LECs. CD200 (also called OX2) is a membrane glycoprotein which binds to the receptor CD200R and has been suggested to act as an immunosuppressor [44,45,46,47]. While *Cd200* was sporadically expressed in the SCS ceiling and floor LECs in the naïve setting, it was up-regulated in the floor during inflammation (Figure 2C). Thus, we next sought to validate the expression and regulation of *Cd200* during inflammation at the protein level. Immunofluorescence staining of murine auricular LNs confirmed CD200 expression in LECs of the SCS ceiling and floor (Figure 2D). Furthermore, FACS analysis of freshly isolated auricular LN LECs confirmed that the expression of CD200 was increased upon imiquimod-induced inflammation in a subset of the cells (Figure 2E,F).

ANXA2 (annexin A2) is a protein involved in diverse cellular processes, such as endocytosis, signal transduction and transcription [48]. While *Anxa2* expression was predominantly found in the ceiling LECs in the steady state as shown before [9], its mRNA level was significantly up-regulated in the floor LECs in inflammatory conditions (Figure 2C). ANXA2 protein expression in LN LECs of the SCS ceiling and floor was also confirmed by immunohistochemistry (Figure 2G). Furthermore, image-based quantification showed a trend towards higher expression in inflamed floor LECs (Figure 2H). 

### 3.4. Floor LECs Display a Distinct RNA “Velocity” Pattern in Inflammation

**Figure 3:**
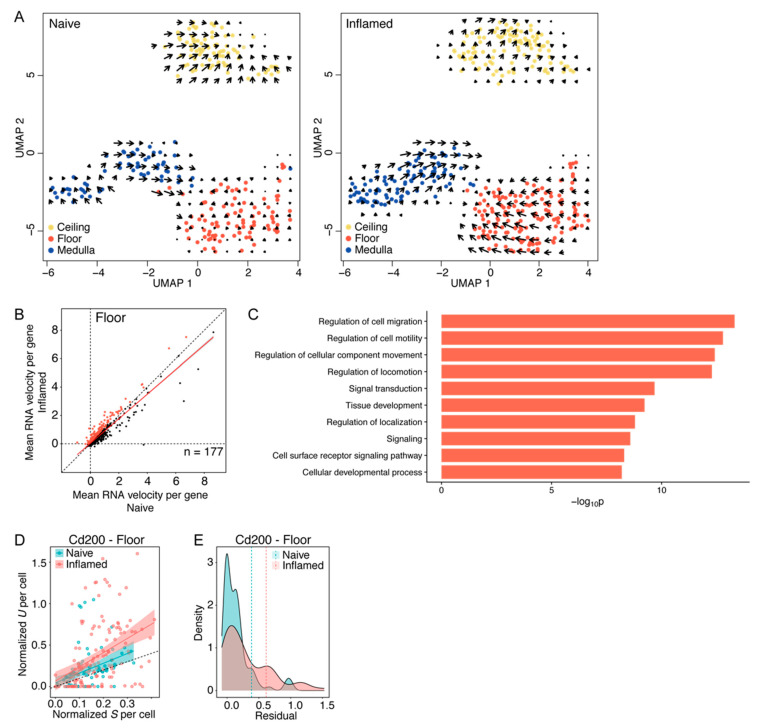
Prediction of future cell states by RNA velocity. (**A**) RNA trajectory of each cell in naïve and inflamed conditions as estimated by Velocyto. (**B**) Correlation of the mean RNA velocity per gene in the floor in inflamed vs. naïve condition. Linear regression is shown in red and the dashed line indicates slope = 1. Selected differentially “speeded” (DS) genes (perpendicular distance to slope = 1 > 0.1) are marked in red. (**C**) Top 10 GO terms of biological processes enriched for DS genes in floor LECs (*p* < 0.5, ranked by *p* values). (**D**) Correlation of spliced (S) and unspliced (U) *Cd200* per cell. Linear regressions for naïve and inflamed conditions are shown in blue and red, respectively. The dashed line indicates slope = 1. (**E**) Deviation of the observed abundance of unspliced *Cd200* from the expected value (residual) in naïve and inflamed conditions, with positive values indicating an expected up-regulation. The dashed lines show the mean per condition.

To better understand the dynamics of the transcriptional changes observed in LN LECs, we used an algorithm to estimate RNA “velocity” at the single-cell level based on the balance between spliced and unspliced transcripts, allowing the prediction of future cellular states [32]. Using this approach, we found that the floor LECs, in particular, displayed distinctive dynamics under inflammation versus the naïve state, whereas the trajectories of medullary and ceiling LECs remained largely unchanged (Figure 3A). In line with the DE analysis, these data further indicate that floor LECs reacted most rapidly and vigorously towards inflammation. Next, we selected distinct gene sets for each LN LEC subpopulation displaying a higher RNA “velocity” in the inflamed condition through correlation analysis of RNA velocity in naïve and inflamed samples. This led to the identification of 177, 218, and 265 differentially “speeded” (DS) genes (perpendicular distance > 0.1 from slope = 1) in floor, ceiling, and medullary LECs, respectively, which may therefore increase in expression in the future (Figure 3B, Supplementary Materials Figure S3A and Table S3). In addition, GO analysis of these DS genes indicated an enrichment in pathways related to signaling responses and transcriptional regulation of the floor and ceiling LECs, biological processes related to cell adhesion, motility, and migration for all three LN LEC subtypes, as well as cell surface receptor signaling for floor and ceiling LECs (Figure 3C and Supplementary Materials Figure S3B). There was a very modest overlap between the sets of DE and DS genes for all LN LEC subpopulations: nine genes in floor LECs, eight genes in ceiling LECs, and none in medulla LECs. Interestingly though, among these we again found *Cd200* that we had validated at the protein level. In fact, the abundance of unspliced *Cd200* indicates a further increase in gene expression in inflamed floor LECs in the future (Figure 3D,E).

### 3.5. LN LECs Potentially Interact with Distinct Immune Cell Types

**Figure 4:**
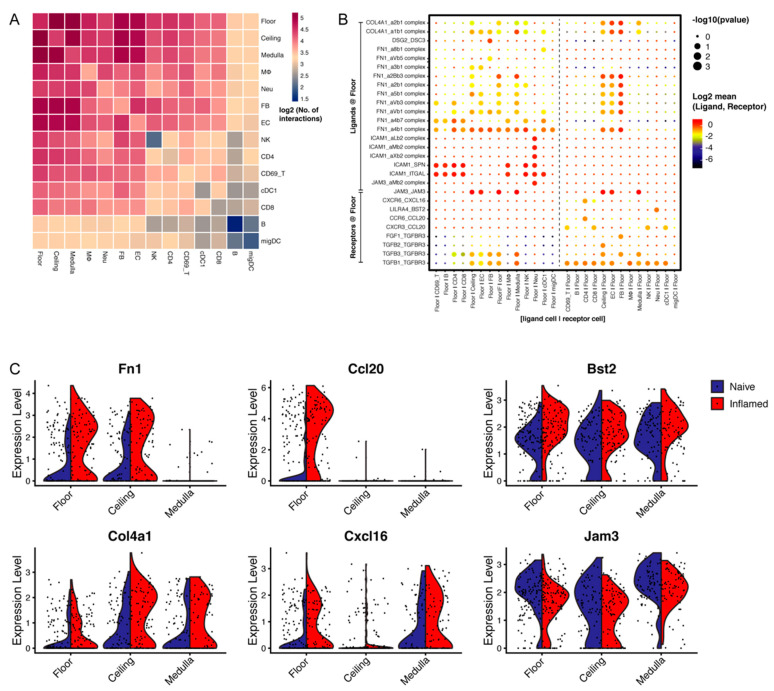
Identification of potential intercellular interactions between LN LECs and other LN cells. (**A**) Heatmap depicting the predicted interactions among different cell populations in naïve LN. The color scale indicates log2 (number of interactions). (**B**) Dot plot of selected interaction pairs involving ligand or receptor molecules that are differentially regulated in floor LECs under inflammation condition over the control. Color gradient is defined by the log2-transformed average expression of ligand and receptor molecules in the interacting cell types, including B cells (B), CD8 T cells, macrophages (MΦ), conventional dendritic cells (cDC1), CD69^+^ T cells (CD69_T), migratory dendritic cells (migDC), NK cells, CD4 T cells, endothelial cells (EC), fibroblasts (FB), and neutrophils (Neu). The circle size denotes −log_10_ (*p* value). (**C**) Example genes that are up- or down- regulated in floor LECs upon imiquimod inflammation.

In addition to interrogating the present and future transcriptional alterations in LN LECs upon inflammation, we also explored the putative crosstalk between LECs and other cell populations residing in naïve LNs by prediction of cell–cell interactions. To this end, we integrated our LN LEC data derived from control mice with publicly available datasets that profiled various LN stromal and immune cell types (Supplementary Materials Figure S4A) [33]. Our interaction analysis identified a multitude of potential ligand–receptor pairs that could mediate their crosstalk (Figure 4A). Notably, certain ligand or receptor molecules were also found to be up- or down-regulated in the floor LECs during imiquimod-mediated inflammation, including extracellular matrix constituents *Fn1* and *Col4a1* (Collagen Type IV Alpha 1 Chain), chemokines *Ccl20* and *Cxcl16*, *Bst2* (bone marrow stromal cell antigen 2) and *Jam3* (junctional adhesion molecule 3) (Figure 4B,C). Changes in the levels of such molecules in floor LECs may indicate changes in the interactions with other LN cells during inflammation. In addition, a moderate level of *Cd200r1* was observed in macrophages (Supplementary Materials Figure S4B). Though originally categorized as the macrophage (MΦ) cluster, it came to our notice that this small subpopulation of *Cd200r1*-expressing cells was enriched for plasmacytoid DC markers (*Siglech* and *Bst2*/*Cd317*) (Supplementary Materials Figure S4C). Hence, our data suggest that elevated expression of *Cd200* in floor LECs may allow interactions with, and perhaps inhibition of, plasmacytoid DCs in inflammation.

### 3.6. Imiquimod-Induced Inflammation Elicits Many Transcriptional Changes also Observed after Cutaneous Oxazolone Challenge 

**Figure 5:**
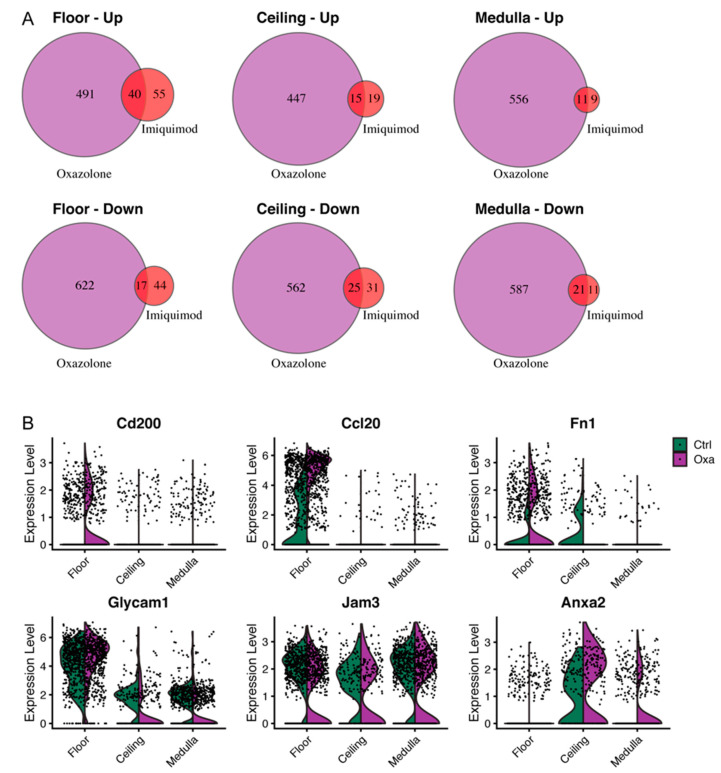
Comparison of dysregulated genes in LN LECs induced by cutaneous oxazolone challenge. (**A**) Overlap of up- or down-regulated genes in different LN LEC subtypes upon inflammation induced by oxazolone or imiquimod. (**B**) Examples of congruently up- (*Cd200*, *Ccl20*, *Fn1*, *Glycam1*) or down-regulated (*Jam3*) genes in floor LECs in the oxazolone dataset.

Repeated imiquimod treatment leads to a psoriasis-like skin inflammation, and the experiments presented here were performed at a time when inflammation had been well established (7 days). A recent scRNA-seq study, which exploited a more acute (48 h) inflammation model using topical oxazolone, illustrated rapid transcriptomic remodeling of LN LECs [10]. Oxazolone as a hapten leads to contact hypersensitivity, eliciting an adaptive immune response with generation of specific T cells [49]. To define the similarities as well as differences in the LN LEC transcriptomes under these conditions, we compared the responses of the three major LN LEC subsets upon imiquimod or oxazolone challenge by re-analyzing and comparing the DE genes identified in that study with ours. LEC subtypes lining the LN sinuses, namely the floor, ceiling and medullary LECs, were defined in accordance with their marker expression in the oxazolone dataset (Supplementary Materials Figure S4D). Intriguingly, around 50% of the dysregulated genes identified in the imiquimod-driven inflammation model were congruently found in the oxazolone dataset (Figure 5A, Supplementary Materials Table S4). Interestingly, we found a prominent elevation in the expression of *Cd200* and *Ccl20*, as well as a further up-regulation of *Glycam1* and downregulation of *Jam3* expression, specifically in the floor LECs upon acute inflammation induced by oxazolone (Figure 5B). In contrast, there was no elevated expression of *Anxa2* in oxazolone-inflamed floor LECs. This indicates that there are considerable similarities in the responses of LN LECs to different types of skin inflammation.

In summary, our results uncover alterations of individual LN LEC subtypes during imiquimod-induced inflammation, pinpointing SCS floor LECs that represent the major site of cell entry into the LN parenchyma from the afferent lymph as the most dynamically responding cells [4,36,50,51].

## 4. Discussion

The structural and anatomical organization of LNs enables efficient antigen delivery and lymphocyte activation in specific areas to optimally trigger adaptive immune responses. In line with the overall spatial organization and cellular specialization in LNs, LECs lining the lymphatic sinuses are heterogenous and fulfill distinct functions. The discovery of specific differences between SCS ceiling (LYVE1^−^, ACKR4^+^) and floor (LYVE1^+^, ACKR4^−^) LECs has fostered great interest in the delineation of LN LEC subtypes and their responses under pathological conditions [37]. Thanks to significant advances in single-cell profiling technologies, there is nowadays a much more comprehensive overview of cellular heterogeneity in LNs, including diverse immune cell populations as well as stromal cells [10,33,52,53,54,55]. Considering the functional disparities among subpopulations of LN LECs, molecular characterization of their heterogeneity allowed us to dissect individually dynamic changes during inflammatory responses and to provide a comprehensive dataset of gene expression in LN LECs in naïve condition versus psoriasis-like skin inflammation. These data clearly show that three major subsets of LN LECs, located at the SCS ceiling, floor and the medullary sinuses, react to inflammation in specific ways.

The imiquimod-driven inflammation model we employed mimics many features of psoriasis at the histological and transcriptional levels [17,18]. Many studies, based on both mouse models and psoriasis patient samples, have illustrated alterations in whole skin, keratinocytes, resident lymphoid cells, macrophages, and other cell types, while changes in the LECs lining the draining LNs have not been studied before [56,57,58]. It is worth noting that many LN LEC-subtype markers remained differentially expressed in their respective locations upon inflammation, indicating that the general distinction between LEC subsets is maintained. Furthermore, there was little overlap in the transcriptional responses of different LN LEC subsets to imiquimod-induced inflammation. Both of these findings further corroborate the fact that subpopulations of LN LECs are endowed with specific biological functions [4]. While most T cells enter the LN parenchyma via high endothelial venules, a subset of T cells and other immune cells such as antigen-presenting dendritic cells are transported to the LN via the afferent lymph. These cells first reach the SCS and subsequently traverse the SCS floor into the cortex and inner medulla [50]. LECs lining the SCS floor, therefore, form the first LN interface, engaging with a host of leukocytes in the afferent lymph. Intriguingly, in comparison with the SCS ceiling and medulla, the most dramatic transcriptional changes were observed in the floor LECs in the imiquimod inflammation model. Furthermore, gene ontology analysis of up-regulated genes in the floor LECs highlighted biological pathways related to type I interferon activity, which is a hallmark of imiquimod-induced psoriasis-like skin inflammation [17,59]. None of the LN LECs in our dataset expressed *Tlr7*. Thus, the observed transcriptional changes were likely not induced by a direct effect of imiquimod on the LN LECs, but rather by cytokines released by, e.g., macrophages and dendritic cells that express TLR7 [16,60].

Among the dysregulated genes in inflamed floor LECs, *Cd200* represents a highly interesting candidate with regard to lymphatic function. CD200 has been shown to be widely expressed in a variety of cell types, including lymphoid cells, macrophages, dendritic cells, neurons, and the blood vascular endothelium. CD200 exerts its immunosuppressive function via the interaction with its cognate receptor CD200R to modulate inflammatory responses [45]. Up-regulation of CD200 in blood vascular endothelial cells has previously been reported in inflammation and cancer studies, which is crucial to the suppression of CD200R-expressing myeloid cells [61,62]. Here, we found for the first time that CD200 is also induced in SCS floor LECs in response to imiquimod-induced inflammation, implicating a potential role in interactions with myeloid cells, including antigen-presenting cells, and potentially in resolving inflammation. On the other hand, while expression of the membrane protein ANXA2 in the LNs has previously been found to be confined to the SCS ceiling under naïve conditions [9], we found that the transcriptional level of *Anxa2* in the floor LECs could be induced under imiquimod-driven inflammation. Recent studies have reported that ANXA2 is a modulator of endothelial junctional proteins and that it functions to maintain vascular integrity [63,64]. Along the same line, the tight junction molecule JAM3 (also known as JAM-C) is another important modulator of inflammatory leukocyte recruitment and transmigration. Following our previous findings of the reduced expression of JAM3 in tumor-draining LN LECs, we have herein observed a similar reduction in *Jam3* expression in the floor LECs in the inflammation model, which is in agreement with previous studies suggesting that lower levels of JAM3 are associated with decreased vascular permeability [15,65,66].

A recent study surveying inflammatory response heterogeneity in mouse cutaneous immune cells upon oxazolone or imiquimod treatment has shed light on the divergent alterations of leukocyte transcriptomes and different molecular signatures underlying the two inflammation models [67]. Likewise, our comparison with a previous scRNA-seq study of LN LECs under a more acute oxazolone inflammation protocol [10] has shown not only similarities but also differences between these two types of skin inflammation. Of note, similar to the imiquimod inflammation model, drastic changes in the floor LECs were also evident upon cutaneous oxazolone challenge. Overall, a stronger reaction of LN LECs was observed in the oxazolone dataset, likely due to the fact that the oxazolone model is more acute and is driven by a T cell-mediated immune response, whereas imiquimod-induced inflammation develops more gradually and is initiated by an innate immune response. Furthermore, despite the discrepancies in the oxazolone and imiquimod scRNA-seq experiments (e.g., type of inflammation, duration, isolation protocols, single-cell platforms, etc.) [68], we found a noticeable overlap between the DE genes in both studies. These genes, including *Glycam1* that was up-regulated in floor LECs and might be involved in leukocyte adhesion [69], thus represent common mediators of LN LEC responses to psoriasiform skin inflammation and contact hypersensitivity.

As many of the dysregulated genes have been associated with immune cell interactions, we searched for putative crosstalk between LECs and various types of LN cell populations and identified potential ligand–receptor pairs mediating such crosstalk in the naïve condition. Potential intercellular interactions were predicted based on the ligand–receptor pairs embodied and curated in the CellPhoneDB repository [35]. While CXCL9, CXCL10 and CXCL11 are commonly known as the cognate chemokines for the receptor CXCR3, the predicted CXCR3–CCL20 pairing (involving the floor LECs in our study) was first reported by Weng et al. in vitro and annotated by IUPHAR/BPS [70]. Though alterations in the level of ligand or receptor molecules in LN LECs may be indicative of an enhanced or suppressed interaction in inflammation, functional experiments or better characterization of the LN immune cell populations in the imiquimod-driven inflammation setting is required to validate these findings.

As compared to previous scRNA-seq studies where additional LN LEC subtypes had been identified (e.g., *Marco^+^* medullary LECs and *Ptx3^+^* cortical LECs) [9,10], we likely analyzed too few LN LECs in the present study to identify these minor subsets. Nonetheless, our data largely captured all the subpopulations of LN LECs as previously described, in terms of the marker genes indicating their spatial organization within murine LNs. Notably, we examined auricular LNs in our sequencing study, as opposed to inguinal, axillary and brachial LNs profiled in other studies, thus providing further evidence that these markers denoting individual LEC subset are consistent between LNs from different anatomical locations. Furthermore, analogous clusters have also been described in an scRNA-seq study of LECs constituting human head, neck, and axillary LNs, indicating that these findings from mouse studies may also be translatable to humans [8].

Taken together, our study provides insights into the specific changes that individual subtypes of murine LN LECs undergo during inflammation at a single-cell resolution and highlights the most drastic transcriptional changes of the SCS floor relative to the ceiling and the medullary sinus LECs. It will be of great interest to investigate in future studies the similarities and differences in the responses of LN LECs to skin inflammation and other pathological conditions, such as cancer or neuroinflammation, and to functionally analyze commonly or distinctively regulated genes.

## Data Availability

Single-cell raw data are available at ArrayExpress under accession number E-MTAB-10435.

## Supplementary Materials

The following are available online at https://www.mdpi.com/article/10.3390/cells10061371/s1: Figure S1: Sorting of LN LECs, confirmation of LEC identity and cluster abundance; Figure S2: Differential gene expression in LN LECs in inflammatory conditions; Figure S3: RNA velocity and differentially “speeded” (DS) genes in ceiling and medullary LECs; Figure S4: Expression of Cd200r1 in the naïve LN and LN LEC marker expressions in the oxazolone dataset; Table S1: Genes differentially expressed between naïve and inflamed conditions in all LEC subsets; Table S2: Gene ontology biological process terms enriched for DE genes between naïve and inflamed conditions; Table S3: Genes differentially “speeded” between naïve and inflamed conditions in all LEC subsets; Table S4: Overlap of differentially expressed genes in the imiquimod and oxazolone studies.
