# Supplementary material for: Single-Cell Transcriptional Heterogeneity of Lymphatic Endothelial Cells in Normal and Inflamed Murine Lymph Nodes
Source: Cells. 2021 Jun 2;10(6):1371. doi: 10.3390/cells10061371 (PMC8229892; doi:10.3390/cells10061371)
Supplement: Supplementary file 1 [file cells-10-01371-s001.zip › Supplementary-Figures.pdf]

Supplementary Figure S1

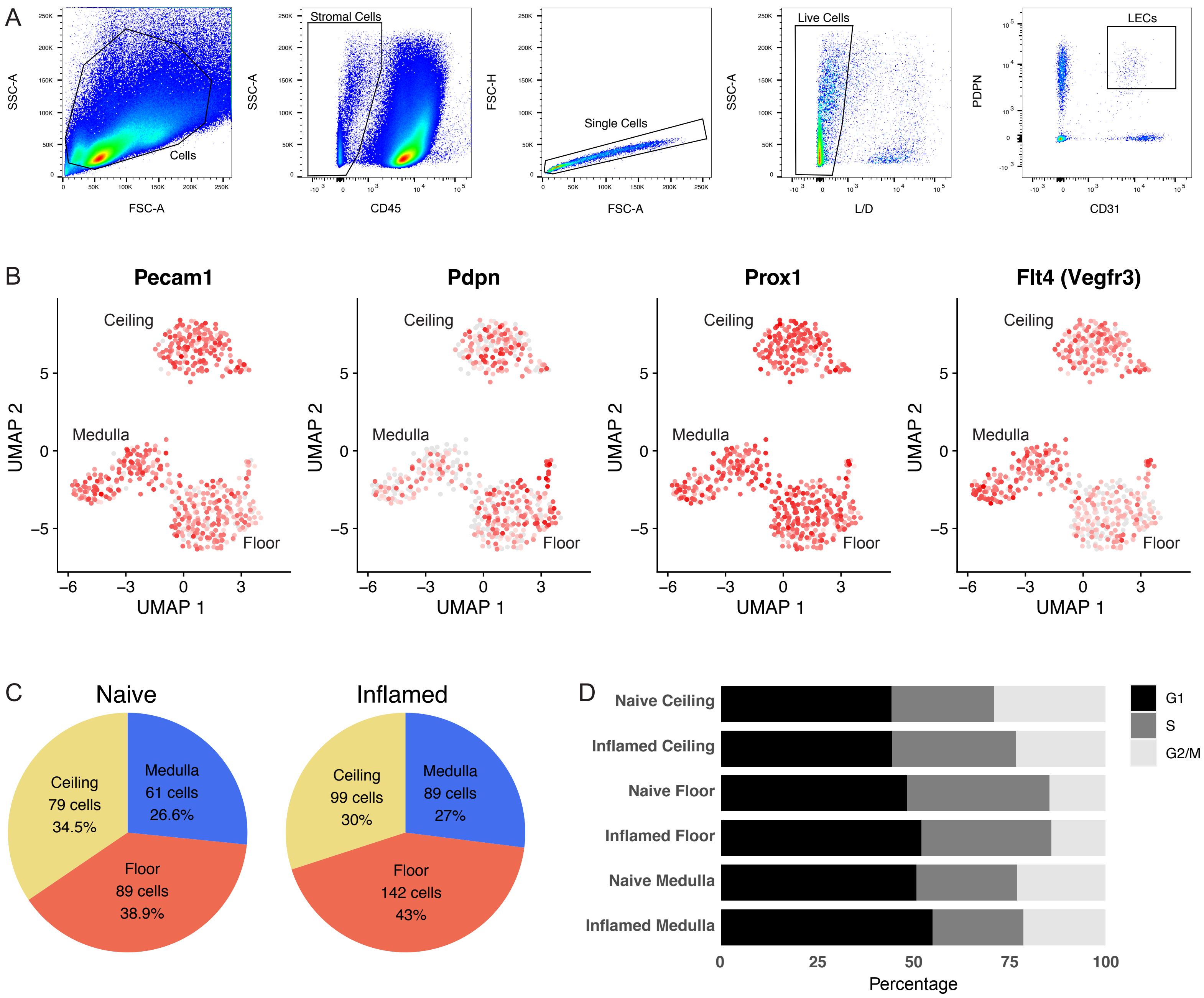

Supplementary Figure S1. Sorting of LN LECs, confirmation of LEC identity and cluster abundance. (A) Sorting strategy for the isolation of single live $CD45^{-}CD31^{+}PDPN^{+}$ LN LECs by FACS. (B) Expression of typical LEC marker genes. (C) Proportions of floor, ceiling and medullary LEC subsets in naïve and inflamed conditions. (D) Percentage of cells in the cell cycle phases G1, S, or G2/M as identified by Seurat for each cluster and condition.

Supplementary Figure S2

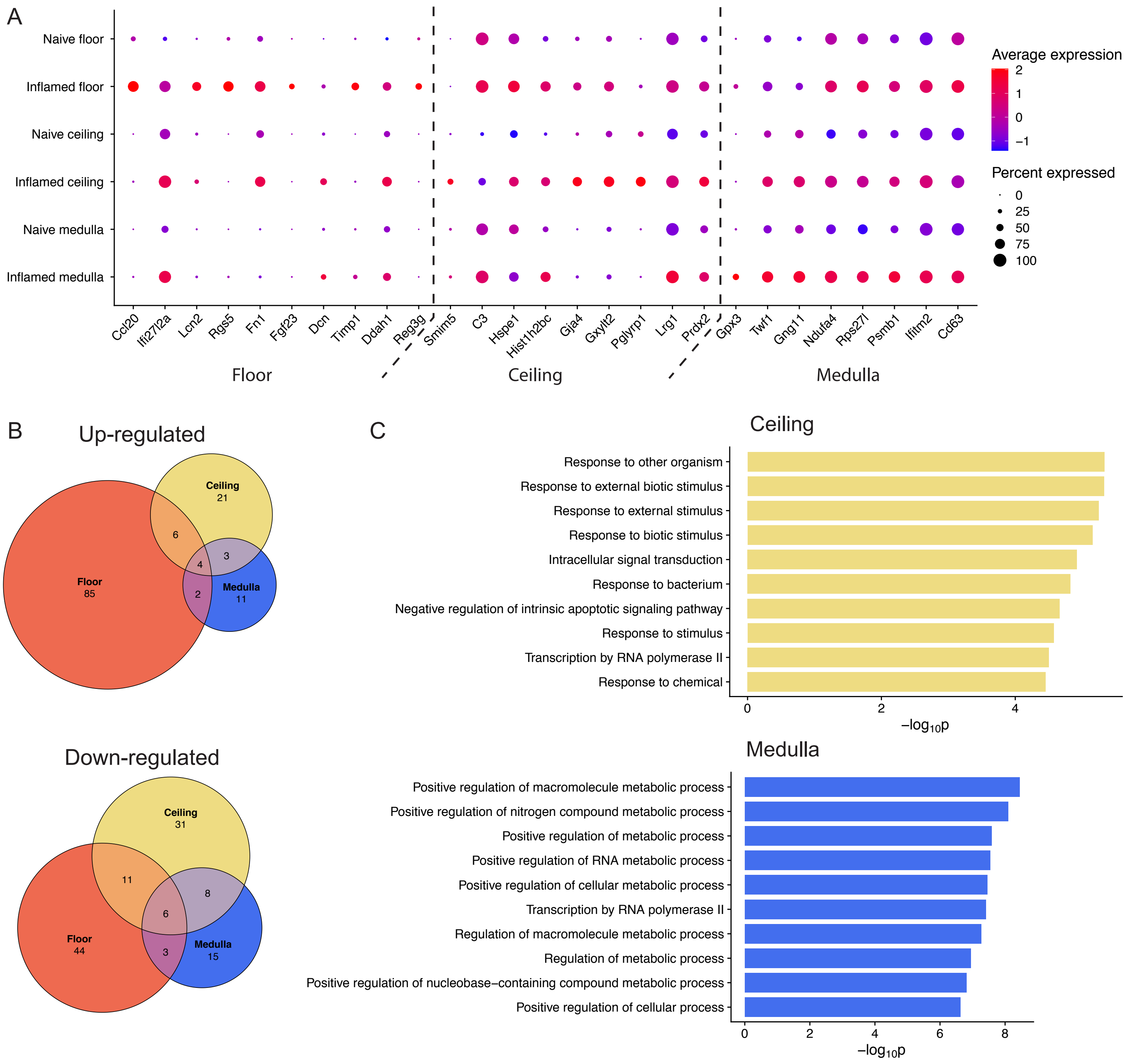

Supplementary Figure S2. Differential gene expression in LN LECs in inflammatory conditions. (A) Dot plot of the top 10 differentially expressed genes ($\log_2$FC > 0.2 and adjusted $p < 0.05$) in each cluster ranked by adjusted p values. Ifi267l2a is a shared DE gene of all three clusters and Lrg1 is a shared DE gene of both ceiling and medulla. (B) Venn diagrams showing overlap of the DE genes in the three clusters. (C) Top 10 GO terms of biological processes enriched for genes differentially expressed in inflammation in ceiling (top) and medullary (bottom) LECs ($p < 0.05$, ranked by p values).

Supplementary Figure S3

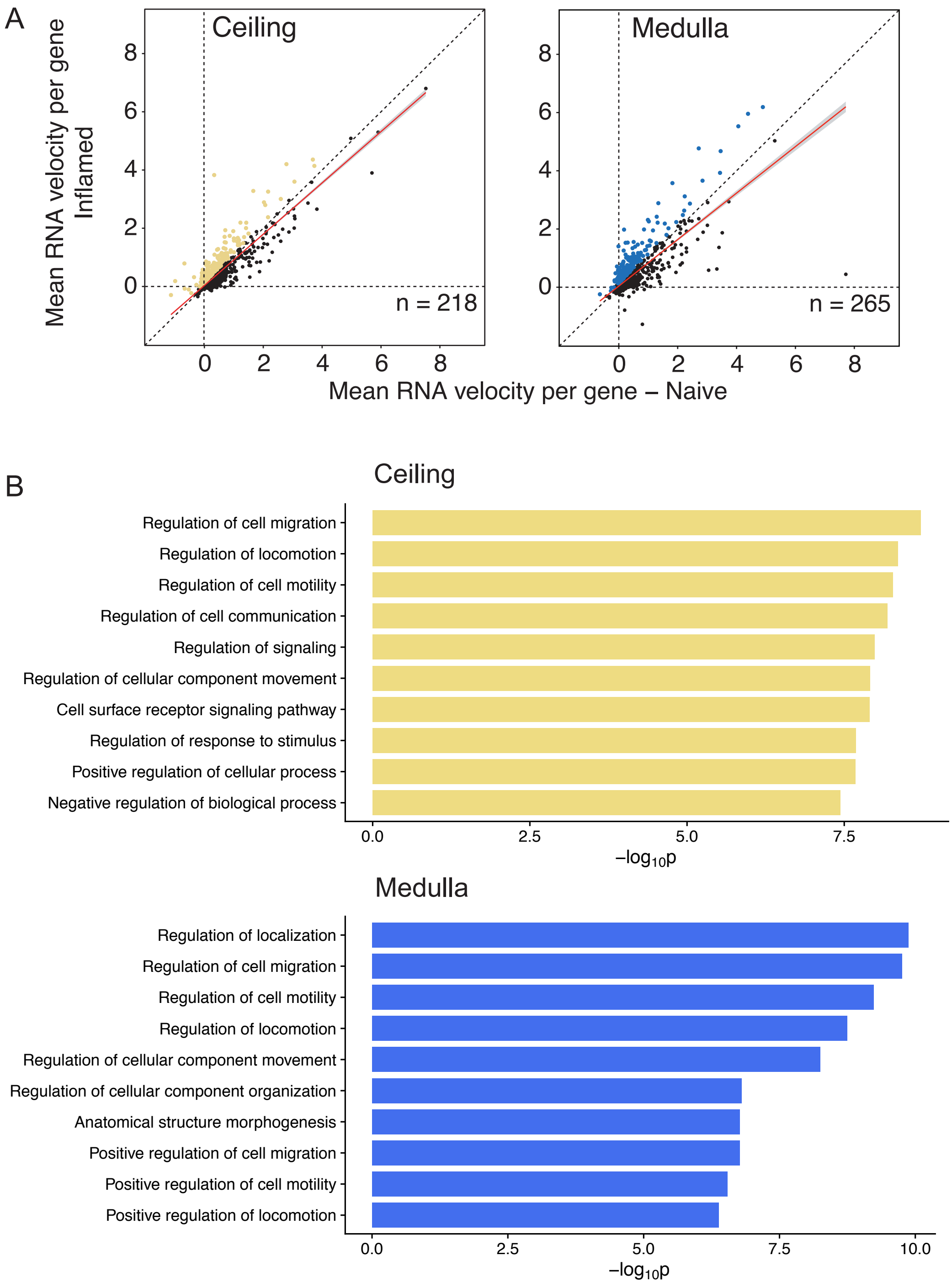

Supplementary Figure S3. RNA velocity and differentially "speeded" (DS) genes in ceiling and medullary LECs. (A) Correlation of the mean RNA velocity per gene in the ceiling (left) and medulla (right) in inflamed vs. naïve condition. Linear regression is shown in red and the dashed line indicates slope = 1. Selected differentially "speeded" (DS) genes are marked in yellow (ceiling LECs) or blue (medullary LECs). (B) Top 10 GO terms of biological processes enriched for DS genes in ceiling (top) and medullary (bottom) LECs ($p < 0.5$, ranked by p values).

Supplementary Figure 4

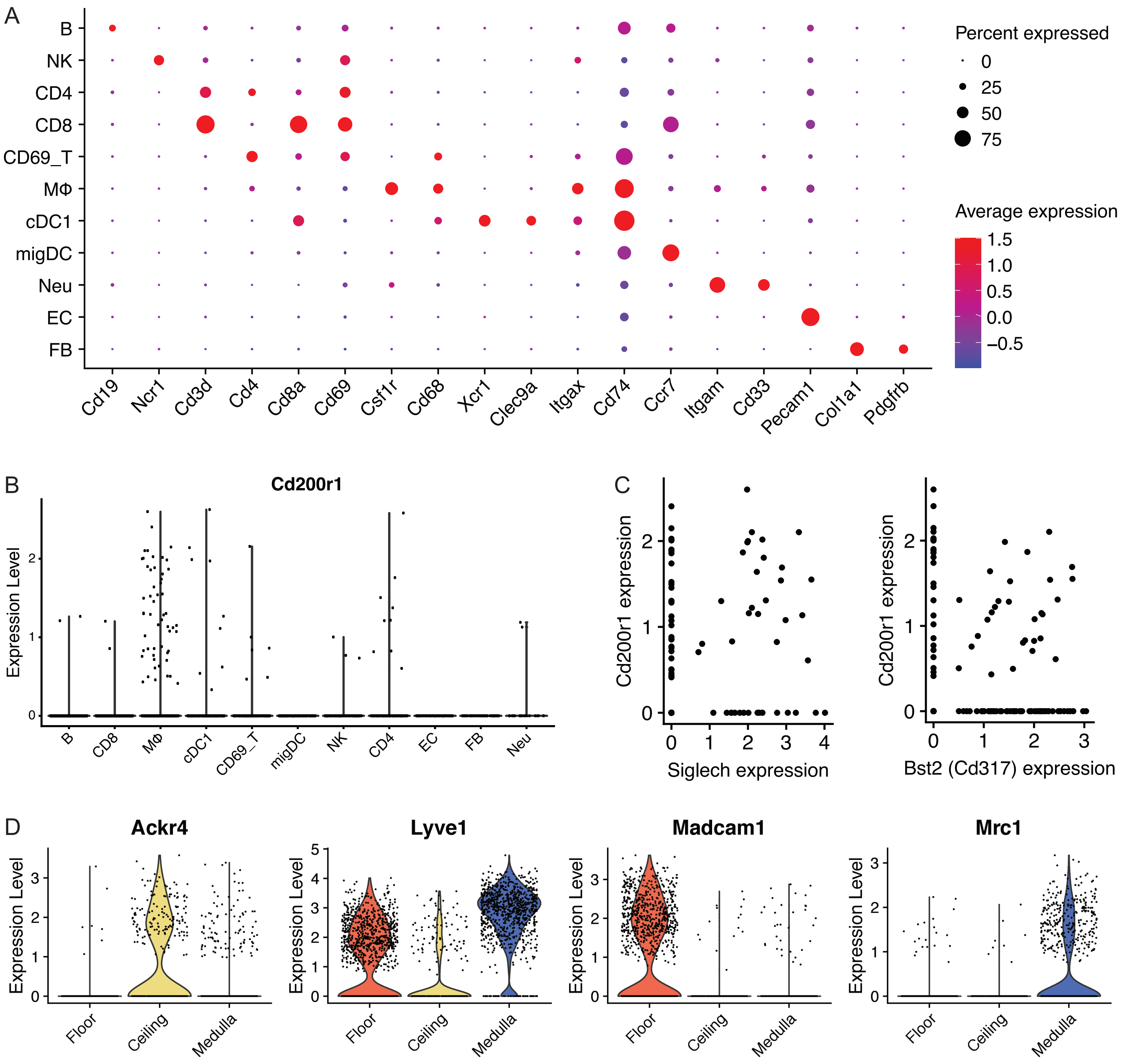

Supplementary Figure S4. Expression of Cd200r1 in the naïve LN and LN LEC marker expressions in the oxazolone dataset. (A) Marker genes denoting different LN immune cell types. (B) Cd200r1 expression in different immune cell populations and stromal cells in naïve LNs. (C) Correlation of Cd200r1 expression with the level of Siglech or Bst2 (Cd317) in the macrophage (MΦ) cluster. (D) Marker expressions in different LN compartments in the oxazolone dataset.
